# The impact of emergency intubation on surgical outcomes in patients with acute aortic dissection AADA (DeBakey I). Is a prompt treatment justified?

**DOI:** 10.1186/s13019-026-04349-6

**Published:** 2026-06-09

**Authors:** Morsi Arar, Erik Beckmann, Andreas Martens, Florian Helms, Heike Krueger, Linda Rudolph, Ezin Deniz, Stefan Todorov, Jan Schmitto, Bastian Schmack, Arjang Ruhparwar, Malakh Shrestha, Tim Kaufeld

**Affiliations:** 1 Department of Cardiothoracic, Transplant and Vascular Surgery, Hanover Medical School, Carl-Neuberg Strasse 1, Hanover, 30625 Germany; 2 Minneapolis Heart Institute at Abbott Northwestern Hospital, Minneapolis, USA; 3 Clinic for Cardiac Surgery, University Clinic Oldenburg, Oldenburg, Germany; 4 Division of CV Surgery, Mayo Clinic, Rochester, MN USA

## Abstract

**Introduction:**

An acute aortic dissection Stanford type A (AADA) is a life-threatening disease and one of the urgent emergencies in cardiovascular surgery. Furthermore, mortality increases when further risk factors like malperfusion, cardiac tamponade or preoperative cardiopulmonary resuscitation (CPR) occur. This study retrospectively evaluates the impact of emergency intubation, performed prior to hospital admission, on early and long-term surgical outcomes in patients with acute aortic dissection Stanford type A (AADA, DeBakey Type I).

**Methods:**

Between January 2000 and January 2018, 430 patients received aortic surgery due to an acute aortic dissection type A (DeBakey Type I) at our tertiary referral hospital. These patients were included in this study. The primary objective was to compare 30-day mortality and long-term survival between intubated and non-intubated AADA patients, while also analyzing differences in preoperative risk factors and postoperative complications. A retrospective analysis with follow-up was conducted.

**Results:**

A minority (*n* = 55; 12.79%) of the entire cohort (*n* = 430) presented themselves in an intubated status and were assigned to Group A, whereas 375 patients were not intubated prior to surgery (Group B). The median age of the entire cohort of 430 patients was 63.7 years and 67.2% patients were male. Group A had a significantly higher number of patients with pericardial tamponade (Group A 58.2%; Group B: 34.7%) and an increased demand for mechanical resuscitation due to pulseless electrical activity (Group A: 29.1%; Group B: 5.9%). Preoperative malperfusion (Group A: 43.6%; Group B: 29.6%; p: 0.036) occurred significantly more often in the intubated cohort. Furthermore, Group A showed a higher number of supra-aortic artery dissections as well as neurological symptoms. The extent of the surgical treatment was comparable for both intubated and non-intubated patients. A total of 38.2% of the intubated AADA patients did not survive the first 30 days after surgery (Group A: 38.2%; Group B: 19.5%; p: 0.002).

**Conclusion:**

Prehospital intubated AADA patients present severe risk factors for early mortality, including pericardial tamponade, severe malperfusion and preoperative resuscitation. Despite the particularly early mortality rate of 40%, the majority of patients benefitted from prompt surgical treatment. Furthermore, the study presents an acceptable long-term outcome after surviving the initial first year after treatment. We therefore recommend that emergent aortic repair should be offered to intubated patients with AADA.

## Introduction

Acute aortic dissection Stanford type A (AADA) is a highly heterogeneous and life-threatening disease [1, 2]. While many patients present in critical condition, clinical presentations can range from sudden death and hemodynamic collapse to various malperfusion syndromes, and occasionally even more stable or asymptomatic cases. Despite this heterogeneity, AADA is associated with a high mortality rate of around 1–2% per hour in the first days if untreated [3]. The pathogenesis is described as a penetrating blood flow through an intimal entry tear creating a false aortic lumen between the intimal and media layer of the aortic wall, often resulting in severe malperfusion, organ impairment or even aortic rupture. Prompt surgical repair remains the gold standard for treating this disease, but even with emergent surgery, early mortality remains high. When untreated, approximately 50% of AADA patients die within the first 48 h, and 75% within 2 weeks [4, 5].

Previous studies found decreasing chances of survival for patients who arrive at the hospital in a poor condition [6–8]. However, Zhou et al. [9] reported that preemptive intubation prior to surgery can improve patients’ prognosis in the cohort of high-risk patients. In contrast to these results, our standard protocol avoids the early intubation of AADA patients. In our experience, prehospital intubation is associated with either cardiopulmonary decompensation during intubation or, in the worst case, in the further ongoing course. In contrast to these results, our standard protocol avoids the early intubation of AADA patients. In our experience, prehospital intubation is associated with either cardiopulmonary decompensation during intubation or, in the worst case, in the further ongoing course.

We thus hypothesize that prehospital intubated patients with AADA (DeBakey I) belong to the most compromised and fragile group in the cohort of AADA patients. Furthermore, the extent of surgery must take into account these critical conditions.

There are only a limited number of publications on this topic and the existing data are inconsistent, with little consensus on the surgical therapy of intubated AADA patients. Our single-center study was designed to evaluate the outcome for patients who received intubation prior to surgical aortic repair of an AADA.

## Patients and methods

### Ethical statement

The institutional ethics committee of the Hannover Medical School approved this retrospective single-center study.

### Study population & inclusion criteria

Between January 2000 and January 2018, a total of 430 patients (67.2% male; 63.7 years (y) median age; interquartile range (IQR) 53.6–71.4 y) received aortic surgery due to an AADA (DeBakey Type I) at our tertiary referral hospital and were included in our study. Main inclusion criteria was a DeBakey I dissections. All patients were comers. The indication was determined by the emergency physician, according to the German guidelines of airway management (DGAI). In all cases, the attending emergency physician performed intubation prior to admission, as further detailed in the ‘Definitions and Intubation Criteria’ subsection.

Patients with iatrogenic dissections, DeBakey Type II and III, as well as chronic dissections, were excluded to prevent further selection bias and to maintain a homogeneous cohort focusing on the most acute and complex Stanford Type A dissections with potential for arch involvement and distal malperfusion, which are key variables in risk stratification scores like GERAADA.

**Inclusion criteria**:

- Aortic dissection DeBakey I (*n* = 430).

**Exclusion criteria**:

- Aortic dissections DeBakey II.
- Chronic dissections.
- Iatrogenic dissections.
- Loss of follow - up.

To evaluate our hypothesis, the cohort was categorized into two subgroups: Group A, comprising AADA patients that were intubated prior to surgery, and Group B, comprising AADA patients that were not preoperatively intubated. Data were collected in our outpatient clinic and patients were seen frequently. In addition, our study nurse team actively contacted patients. Follow-up data collection ended and was completed in February 2022. Patients’ characteristics are presented in Table 1.

**Table 1:** Patients’ characteristics

Characteristics	Entire cohort	Intubated Group (A)	Non-intubated	*P*-value
Total patients	*n* = 430	*n* = 55	*n* = 375	
Age at surgery (years), median (IQR)	63.7 (53.6–71.4)	60.8 (51.4–71.4)	63.9 (53.7–71.5)	0.502
Sex male, n (%)	289 (67.2)	34 (61.8)	255 (68.0)	0.362
BMI, median (IQR)	26.2 (24.2–29.1)	26.5 (24.2–30.5)	26.1 (24.2–28.4)	0.145
Hypertension, n (%)	278 (64.7)	34 (61.8)	244 (65.1)	0.652
Diabetes mellitus, n (%)	30 (7.0)	6 (10.9)	24 (6.4)	0.252
Pvod, n (%)	19 (4.4)	1 (1.8)	18 (4.8)	0.490
COPD, n (%)	40 (9.3)	8 (14.5)	32 (8.5)	0.152
Coronary heart disease, n (%)	46 (10.7)	3 (5.5)	43 (11.5)	0.178
Hyperthyreodism, n (%)	3 (0.7)	1 (1.8)	2 (0.5)	0.337
Hypothyreodism, n (%)	36 (8.4)	3 (5.5)	33 (8.8)	0.601
Atrial fibrillation, n (%)	53 (12.3)	5 (9.1)	48 (12.8)	0.435
Marfan syndrome, n (%)	19 (4.4)	2 (3.6)	17 (4.5)	1.000
Pericardial tamponade, n (%)	162 (37.7)	32 (58.2)	130 (34.7)	< 0.001
Bicuspid aortic valve, n (%)	21 (4.9)	1 (1.8)	20 (5.3)	0.498
Mechanical resuscitation, n (%)	38 (8.8)	16 (29.1)	22 (5.9)	< 0.001
Cardiac re-operation, n (%)	15 (3.5)	2 (3.6)	13 (3.5)	1.000
Malperfusion, n (%)	135 (31.4)	24 (43.6)	111 (29.6)	0.036
Cerebral malperfusion, n (%)	49 (11.4)	10 (18.2)	39 (10.4)	0.090
Visceral malperfusion, n (%)	36 (8.4)	11 (20.0)	25 (6.7)	0.003
Renal malperfusion, n (%)	49 (11.4)	14 (25.5)	35 (9.3)	< 0.001
Limb malperfusion, n (%)	61 (14.2)	10 (18.2)	51 (13.6)	0.363
Hemiparesis, n (%)	26 (6.0)	8 (14.5)	18 (4.8)	0.011
Seizure, n (%)	7 (1.6)	2 (3.6)	5 (1.3)	0.222
Evidence of stroke CT, n (%)	26 (6.0)	4 (7.3)	22 (5.9)	0.760
Neurologic symptoms, n (%)	84 (19.5)	24 (43.6)	60 (16.0)	< 0.001
Dissection supra-aortic arteries, n (%)	88 (20.5)	17 (30.9)	71 (18.9)	0.040
Dissection LCA, n (%)	12 (2.8)	3 (5.5)	9 (2.4)	0.189
Dissection RCA, n (%)	42 (9.8)	8 (14.5)	34 (9.1)	0.222
Painful event prior to surgery (h), median (IQR)	6.0 (4.0–12.1)	6.0 (4.0–12.0)	6.0 (4.0–14.0)	0.456

IQR (interquartile range), BMI (body mass index), PVOD (peripheral vascular occlusion disease), COPD (chronic obstructive pulmonary disease), CT (computer tomography), LCA (left coronary artery), RCA (right coronary artery)

### Definitions and intubation criteria

“Prehospital intubation” was defined as any procedure performed by the emergency physician team prior to arrival at our tertiary center. This includes intubations performed at the scene, during transport, or at a referring hospital before transfer to our center. Patients intubated upon arrival at our own Emergency Department or operating room were categorized into Group B (non-intubated prior to surgery) for the purpose of this study. The indication for intubation was determined by the attending emergency physician on-site based on clinical assessment and in accordance with the S1 guidelines of the German Society of Anesthesiology and Intensive Care Medicine (DGAI). These guidelines typically indicate emergency airway management for a Glasgow Coma Scale (GCS) < 9, acute respiratory insufficiency, or severe hemodynamic instability (typically SBP < 90 mmHg).

### Follow-up

The postoperative follow-up of the patients took place in our outpatient clinic at the following intervals: 3 months postoperatively, 6 months postoperatively and then once a year. When no pathological finding occurred after three years, the patients were invited to the outpatient clinic every two years. Patients were contacted both by telephone call and by letter. We obtained informed consent for the collection of follow-up data. Two patients were lost to follow – up.

### Additional definitions

The existence of a dissection membrane starting in the ascending aorta or an intramural hematoma inside the aortic wall was defined as the radiographic equivalent of an AADA. “Intubation” status was fulfilled when the intubation was performed prior to admission to our hospital.

Patients who presented themselves with severe neurologic symptoms like apraxia, hemiplegia or dysarthria but without a cerebral CT scan performed prior to surgery and postoperative evidence of stroke were assigned to the preoperative stroke group. A stroke had to be verified by CT or magnetic resonance imaging (MRI). We used the classification of Sievers et al. [10] for the definition of malperfusion (TEM aortic dissection classification stage M2 and M3 ((−), (+)). The diagnosis of a dissection of the coronary arteries was verified either using coronary angiography or by being visible intraoperatively. AADAs accidently induced during open-heart surgery were defined as iatrogenic dissection and were excluded from this study. Dissections detected postoperatively using CT or MRI were defined as persisting dissections. For the diagnosis of hypertension, diabetes mellitus or chronic obstructive pulmonary disease (COPD), a preoperatively performed medical treatment was mandatory.

### Specifically for neurological symptoms & radiographic imaging

- Cerebral Malperfusion: Radiographic evidence of at least one carotid- or vertebral artery.
- Hemiparesis: Unilateral motor weakness on clinical examination.
- Seizure: Witnessed abnormal electrical activity or motor movements.
- Neurologic symptoms: A collective term including altered mental status, apraxia, or dysarthria.
- Evidence of stroke CT (Table 1): Radiographic evidence of stroke on computed tomography (CT) performed **preoperatively**.
- CCT Stroke (Table 3): A collective term for pre- and postoperatively detected strokes using computed tomography (CT).
- New-onset stroke (Table 3): Postoperatively radiographic evidence of stroke, without preoperative occurrence of neurological symptoms.

### Surgical methods

Perioperative management and surgical technique has been previously described by our group [11]. Our department provides emergency medical service to a population of around two million citizens. The distance for helicopter- and ground-based patient transfer is a maximum of 100 km.

In order to avoid early cardiac decompensation, we aim to perform intubation once all anesthesiological and surgical preparations have been completed. Intubation and the establishment of full sternotomy extra-corporal circulation (ECC) follows this. Our cannulation technique in cases of AADA has been previously published by our group [12]. Furthermore, in 2010 we began using the beating heart technique for cardio protection in cases of extended arch surgery [13].

Our department was one of the first centers to implement an FET treatment in cases of AADA. From 2000 to 2010, the FET technique was performed using a custom-made Chavan-Haverich prosthesis followed by a prefabricated Chavan-Haverich hybrid graft [14] (Curative GmbH, Dresden, Germany). From 2005 to 2010, the Jotec E-vita hybrid graft was used [15]. In 2007 we changed our strategy from a straight graft with island technique to the branched Sienna™ graft (Terumo^®^, Glasgow, UK), even for total or hemi-arch replacement. In 2010 we switched to the four-branched frozen elephant trunk prosthesis (FET Vascutek Terumo, Terumo^®^, Glasgow, UK) [16].

Proximal aortic arch replacement is characterized by the distal anastomosis being located proximal to the brachiocephalic trunk. In a proximal aortic arch replacement, no supra-aortic arteries are re-implanted into the prosthesis. A subtotal arch replacement involves an additional reimplantation brachiocephalic trunk. Limited aortic repair in terms of a proximal arch replacement was performed using established straight Dacron grafts.

### Statistical analysis

For data analysis, SPSS Statistics 27 software (IBM Corp. released 2020; IBM SPSS Statistics for Windows, Version 27.0; Armonk, NY: IBM Corp.) was used. A normal distribution of variables was calculated using the Kolmogorov–Smirnov test. Categorical variables are stated as absolute numbers (n) and proportions. Normally distributed continuous variables are stated as mean ± standard deviation, while continuous variables without normal distribution are stated as median and IQR. Fisher’s exact test was used to detect differences in categorical variables. Differences in continuous variables were tested using the Mann-Whitney U test. Kaplan–Meier analysis and log rank were used for the evaluation of survival, and the log rank test was used to test for differences. We did not correct for multiple testing. A value of *p* < 0.05 was considered statistically significant.

## Results

### Patient demographics

The preoperative patient demographics are shown in Table 1. The minority (*n* = 55; 12.79%) of the entire cohort (*n* = 430) presented themselves in an intubated status and were assigned to Group A; 375 patients were not intubated prior to surgery. The median age of the entire cohort of 430 patients was 63.7 years (Group A: 60.8 y; Group B 63.9 y; p: 0.502) and 67.2% patients (Group A: 61.8%; Group B: 68%; p: 0.362) were male. Concomitant diseases like COPD (Group A: 14.5%; Group B: 8.5%; p: 0.152) and diabetes mellitus occurred more often in Group A without proven significance. The majority of both groups received medical treatment for proven arterial hypertension (Group A: *n* = 34 (61.8%); Group B: *n* = 244 (65.1%); p: 0.652). There were no significant differences in the medical history between the cohorts. In contrast, patients’ health conditions differed significantly at admission. Group A had a significantly higher number of pericardial tamponade (Group A: *n* = 32 (58.2%); Group B: *n* = 130 (34.7%); p: <0.001). Furthermore, there was an increased demand for mechanical resuscitation due to pulseless electrical activity in the intubated group (Group A: *n* = 16 (29.1%); Group B: *n* = 22 (5.9%); p: <0.0001).

Our results show fundamental differences in terms of the preoperative existence of malperfusion and neurological disabilities. Cerebral malperfusion (Group A: *n* = 10 (18.2%); Group B: *n* = 39 (10.4%); p: 0.090), visceral malperfusion (Group A: *n* = 11 (20.0%); Group B: *n* = 25 (6.7%); p: 0.003) and renal malperfusion (Group A: *n* = 14 (25.5%); Group B: *n* = 35 (9.3%); p: <0.001) occurred more often in Group A. An increased rate of patients with preoperative hemiparesis (Group A: *n* = 8 (14.5%); Group B: *n* = 18 (4.8%); p: 0.011) and neurological symptoms (Group A: *n* = 24 (43.6%); Group B: *n* = 60 (16.0%); p: <0.001) correlate with a significantly higher number of supraaortic artery dissections (Group A: *n* = 17 (30.9%); Group B: *n* = 71 (18.9%); p: 0.040). Preoperatively evidenced strokes were equally distributed in both groups (Group A: *n* = 4 (7.3%); Group B: *n* = 22 (5.9%); p: 0.760).

### Intraoperative data

Detailed intraoperative data are shown in Table 2. Total operation time was not significantly shorter in the intubated cohort (Group A: 307.0 min (252.0–372.0); Group B: 335.0 min (260.0–409.0); p: 0.163). Neither cardiopulmonary bypass time (Group A: 215.0 min; Group B: 218.0 min; p: 0.579) nor aortic cross-clamp time differed significantly between the groups (Group A: 126.0 min; Group B: 126.0 min; p: 0.825). The extent of the surgical treatment was comparable for both intubated and non-intubated patients. Interestingly, our beating heart intraoperative perfusion procedure was preferred in Group B (Group A: *n* = 1 (1.8%); Group B: *n* = 63 (16.8%); p: 0.004).

**Table 2:** Intraoperative data

Characteristics	Entire cohort	Intubated	Non-intubated	*P*-value
Total patients	*n* = 430	*n* = 55	*n* = 375	
Total operation time (min), median (IQR)	330.5 (259.8–404.3)	307.0 (252.0 − 372.0)	335.0 (260.0–409.0)	0.163
Cardiopulmonary bypass time (min), median (IQR)	217.0 (169.5–285.0)	215.0 (159.0 − 263.0)	218.0 (170.0–286.0)	0.579
Aortic cross-clamp time (min), median (IQR)	126.0 (92.8–161.3)	126.0 (91.0 − 158.0)	126.0 (94.0–162.0)	0.825
HCA (hypothermic circulatory arrest) time (min), median (IQR)	36.0 (25.0–52.0)	41.0 (25.0–61.0)	35.0 (25.0–52.0)	0.276
SACP (selective antegrade cerebral perfusion) time (min), median (IQR)	32.5 (19.0–76.0)	32.0 (19.0–52.0)	33.0 (20.0–77.0)	0.254
Minimum core temperature (°C), median (IQR)	24.7 (22.2–26.0)	24.6 (23.0–26.5)	24.7 (22.0–26.0)	0.079
Erythrocyte concentrates, median (IQR)	6.0 (4.0–10.0)	7.0 (4.0–12.0)	6.0 (4.0–10.0)	0.095
Fresh frozen plasma, median (IQR)	6.0 (4.0–10.0)	6.0 (6.0–10.0)	6.0 (4.0–10.0)	0.058
Platelet concentrates, median (IQR)	3.0 (2.0–4.0)	2.0 (2.0–4.0)	3.0 (2.0–4.0)	0.556
Beating heart, n (%)	64 (14.9)	1 (1.8)	63 (16.8)	0.004
Arch replacement:				
Proximal arch replacement, n (%)	192 (44.7)	25 (45.5)	167 (44.5)	0.898
Subtotal arch replacement, n (%)	34 (7.9)	6 (10.9)	28 (7.5)	0.419
Total arch replacement, n (%)	36 (8.4)	5 (9.1)	31 (8.3)	0.796
Total arch replacement elephant trunk, n (%)	47 (10.9)	9 (16.4)	38 (10.1)	0.167
Total arch replacement frozen elephant trunk, n (%)	121 (28.1)	10 (18.2)	111 (29.6)	0.079
Bio glue, n (%)	146 (34.0)	17 (30.9)	129 (34.4)	0.610
Aortic valve replacement:				
Biologic, n (%)	65 (15.1)	4 (7.3)	61 (16.3)	0.082
Mechanic, n (%)	67 (15.6)	11 (20.0)	56 (14.9)	0.333
Root involvement, n (%)	258 (60.0)	32 (58.2)	226 (60.3)	0.768
Bentall, n (%)	129 (30.0)	15 (27.3)	114 (30.4)	0.636
David, n (%)	98 (22.8)	13 (23.6)	85 (22.7)	0.873
Yacoub, n (%)	19 (4.4)	4 (7.3)	15 (4.0)	0.285
CABG, n (%)	77 (17.9)	8 (14.5)	69 (18.4)	0.486
ECMO, n (%)	19 (4.4)	0 (0.0)	19 (5.1)	0.151
Intraoperative mortality, n (%)	12 (2.8)	3 (5.5)	9 (2.4)	0.189

Min (minute), IQR (interquartile range), C° (Celcius), CABG (coronary artery bypass graft), ECMO (Extracorporeal Membrane Oxygenation)

### Postoperative data

The early postoperative outcome is shown in Table 3. Overall survival was reduced, but not significantly, in Group A (Group A: 875.0 d (4.0–3649.0); Group B: 1741.0 d (115.0–3135.0); p: 0.131). Preoperatively intubated patients needed a significant longer ventilation time (Group A: 72.0 h (33.0–172.0); Group B: 43.0 h (19.0–136.0); p: 0.041). Of the intubated AADA patients, 38.2% did not survive the first 30 days after surgery (Group A: *n* = 21 (38.2%); Group B: *n* = 73 (19.5%); p: 0.002).

**Table 3:** Postoperative data

Characteristics	Entire cohort	Intubated	Non-intubated	*P*-value
Total patients	*n* = 430	*n* = 55	*n* = 375	
Survival time (h), median (IQR)	1667.5 (71.0–3212.0)	875.0 (4.0–3649.0)	1741.0 (115.0–3135.0)	0.131
Ventilation time (h)	48.0 (21.0–138.3)	72.0 (33.0–172.0)	43.0 (19.0–136.0)	0.041
Intensive care unit (days), median (IQR)	4.0 (2.0–8.0)	4.0 (2.0–9.0)	4.0 (2.0–8.0)	0.947
Rethoracotomy, n (%)	71 (16.5)	10 (18.2)	61 (16.3)	0.699
Dialysis, n (%)	55 (12.8)	8 (14.5)	47 (12.5)	0.677
30-day mortality, n (%)	94 (21.9)	21 (38.2)	73 (19.5)	0.002
CCT stroke, n (%)	84 (19.5)	15 (27.3)	69 (18.4)	0.121
New-onset stroke, n (%)	37 (8.6)	4 (7.3)	33 (8.8)	1.000
Remaining cerebral malperfusion, n (%)	16 (3.7)	1 (1.8)	15 (4.0)	0.706
Remaining limb malperfusion, n (%)	13 (3.0)	5 (9.1)	8 (2.1)	0.016
Remaining visceral malperfusion, n (%)	10 (2.3)	3 (5.5)	7 (1.9)	0.124
Remaining renal malperfusion, n (%)	20 (4.7)	2 (3.6)	18 (4.8)	1.000

IQR (interquartile range), h (hour), CCT (cranial computed tomography)

Although the overall stroke rate was elevated in Group A (Group A: *n* = 21 (38.2%); Group B: *n* = 73 (19.5%); p: 0.002), both groups showed a comparable incidence of new onset strokes (Group A: *n* = 4 (7.3%); Group B: *n* = 33 (8.8%); p: 1.000). Furthermore, data showed the tendency of postoperatively remaining limb malperfusion (Group A: *n* = 5 (9.1%); Group B: *n* = 8 (2.1%); p: 0.016) and remaining visceral malperfusion (Group A: *n* = 3 (5.5%); Group B: *n* = 7 (1.9%); p: 0.124) in Group A.

### Late outcome

**Fig. 1:**
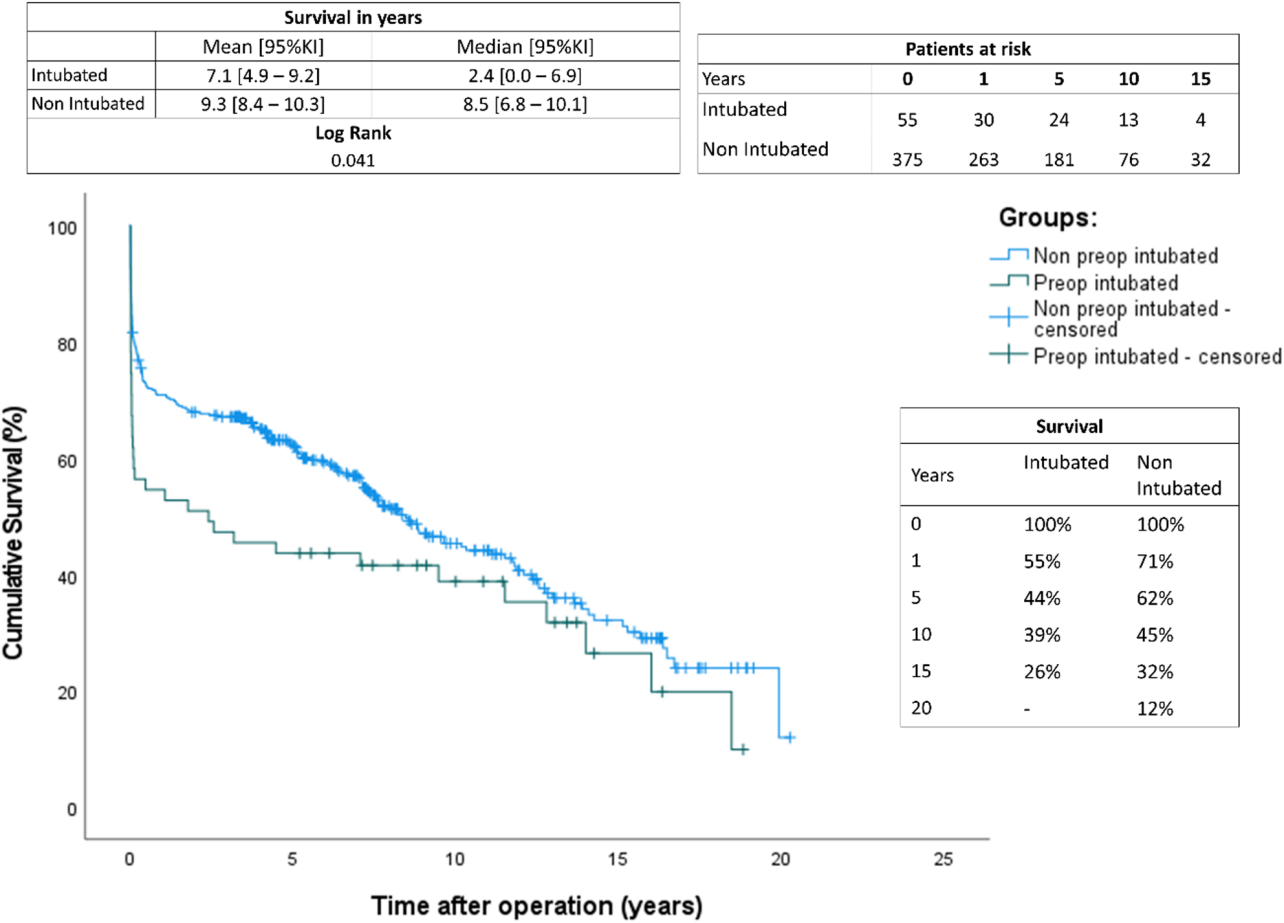
Kaplan-Meier curves represent the survival of intubated vs. non-intubated AADA patients. X-axis denotes time after surgery (years). Y-axis denotes cumulative survival (%)

The Kaplan-Meier survival curves are shown in Fig. 1. Survival after 1, 5 and 10 years was 55%, 44% and 39% in Group A, respectively, and 71%, 62% and 45%, respectively, in Group B. Mean (CI 95%) survival of intubated patients was 7.1 y (4.9–9.2 y), and 9.3 y (8.4–10.3 y) in non-intubated patients.

Rates of aortic re-operations are presented in Table 4. A total of 53 patients (12.3%) received secondary aortic surgery. The rate of aortic re-operations was equally distributed in both groups (Group A: *n* = 7 (12.7%); Group B: *n* = 46 (12.3%); p: 0.923). The same findings were observed in the identical aortic area as well as in the downstream aorta.

**Table 4:** Aortic re-operations

Characteristics	Entire cohort	Intubated	Non-intubated	*P*-value
Total patients	*n* = 430	*n* = 55	*n* = 375	
Secondary aortic operation, n (%)	53 (12.3)	7 (12.7)	46 (12.3)	0.923
Re-operation identical area, n (%)	16 (3.7)	3 (5.5)	13 (3.5)	0.443
Re-operation downstream aorta, n (%)	37 (8.6)	4 (7.3)	33 (8.8)	1.000
TAA repair, n (%)	9 (2.1)	0 (0.0)	9 (2.4)	0.611
Y-prosthesis, n (%)	4 (0.9)	0 (0.0)	4 (1.1)	1.000
Descending repair, n (%)	18 (4.2)	1 (1.8)	17 (4.5)	0.491
Hybrid, n (%)	7 (1.6)	3 (5.5)	4 (1.1)	0.048
TEVAR, n (%)	13 (3.0)	1 (1.8)	12 (3.2)	1.000
EVAR, n (%)	5 (1.2)	0 (0.0)	5 (1.3)	1.000
Aortic fenestration (%)	2 (0.5)	0 (0.0)	2 (0.5)	1.000

TAA (thoracoabdominal repair), TEVAR (thoracic endovascular aortic repair), EVAR (endovascular aneurysm repair)

## Discussion

AADA is a life-threatening event associated with particularly high early mortality despite prompt surgical treatment [17, 18]. Even in critical conditions, additional risk factors such as CPR, malperfusion, or pericardial tamponade may adversely affect the patient’s prognosis.

However, due to the fatal prognosis, the patients’ conditions at admission require an individual evaluation of whether either surgical treatment or even a conservative procedure is recommended.

Ventilation at referral is a known risk factor and is taken into consideration in the German Registry of Acute Aortic Dissection Type A (GERAADA) score for predicting early mortality [19, 20]. The 30-day mortality of 38.2% in our study correlates with the predicted early mortality of 37.1% [20] in cases of prehospital ventilation according to the GERAADA score.

However, patient’s conditions between the groups differed substantially on admission to our hospital. Whether prompt repair is a curative procedure depends on various concomitant risk factors.

### Intubation as a marker of severity

The significantly worse outcomes observed in Group A are highly likely to reflect the severity of the underlying disease—evidenced by higher rates of pericardial tamponade, mechanical resuscitation, and malperfusion—rather than a direct detrimental effect of the intubation procedure itself. This phenomenon, known as ‘confounding by indication,’ is central to interpreting our findings. Intubation in this context is a response to an already critical physiological state. While Zhou et al. [9] discussed ‘preemptive’ intubation as a potentially stabilizing measure in a different clinical context, our cohort consists of patients requiring ‘emergency’ intubation due to acute clinical deterioration. These represent two fundamentally different clinical scenarios: a preemptive strategy aims to prevent collapse, whereas emergency intubation is a response to existing collapse. Therefore, intubation status in our study serves as a powerful surrogate marker for extreme disease severity.

Moreover, surgery should be reconsidered for patients who cannot achieve return of spontaneous circulation after pericardiotomy [7].

According to the GERAADA score there is a predicted early mortality of 37.1% [20] in cases of prehospital ventilation. The 30-day mortality of 38.2% in our study correlates with these data.

However, patient’s conditions between the groups differed substantially on admission to our hospital. Whether prompt repair is a curative procedure depends on various concomitant risk factors.

The hypothesis that intubated patients belong to the vulnerable cohort was upheld. In our study, intubated patients included a significantly higher number of proven malperfusion (A: *n* = 24 (43.6%); B: *n* = 111 (29.6%); p: 0.036) and preoperatively occurring neurological symptoms (A: *n* = 24 (43.6%); B: *n* = 60 (16.0%); p: <0.001). It can be assumed that even the neurological symptoms that are associated with a higher number of dissected supra-aortic arteries (A: *n* = 24 (43.6%); B: *n* = 60 (16.0%); *p* < 0.001) are among the leading indications for non-elective intubation.

Our data also show a significant association between mandatory intubation in AADA patients with a higher incidence of further independent risk factors, like pericardial tamponade (A: *n* = 32 (58.2%); B: *n* = 130 (34.7%); p: <0.001) or preoperative resuscitation (A: *n* = 16 (29.1%); B: *n* = 22 (5.9%); p: <0.001). Our group has previously published data that shows that even this pericardial effusion significantly increases the risk of early mortality [21]. Further studies have found preoperative cardiopulmonary resuscitation to be the main predictor for operative mortality [22].

### Surgical implications and adaptations

Despite the fact that the intubated cohort presented with a variety of severe risk factors, the overall extent of surgical treatment (e.g., total operation time, cardiopulmonary bypass time, aortic cross-clamp time, and arch replacement strategies) was comparable between groups. However, the significantly lower use of the ‘beating heart’ perfusion technique in Group A (1.8% vs. 16.8%; *p* = 0.004) is noteworthy. This difference likely reflects the surgeons’ preference for a more rapid, conventional repair strategy in highly unstable patients to minimize operative time and myocardial ischemia in the face of extreme hemodynamic instability. This highlights how surgical decision-making adapts to the patient’s critical preoperative condition, prioritizing speed and established techniques over potentially more complex or time-consuming approaches. In this context, limited surgical repair with associated reduced aortic cross-clamping and cardiopulmonary bypass times must be discussed to avoid additional known risk factors (24).

Interestingly, Yuanxi et al. [23] concluded that safe extubation after 72 h after AADA surgery is crucial for achieving favorable outcomes. In our study, postoperative ventilation time was extended up to exactly 72 h in Group A (Group A: 72 h (33.0–172.0 h); Group B: 43.0 h (19.0–136.0 h); p: 0.041).

Our long-term survival data, demonstrating that the survival difference is most pronounced in the first postoperative year and then becomes comparable between groups (Kaplan-Meier Curve; Fig. 1), further supports the notion that early mortality is largely driven by the severe preoperative compromise necessitating intubation, rather than the intubation itself or the specific surgical strategy. This reinforces our conclusion that emergent aortic repair should be offered to intubated patients with AADA, as survivors can achieve acceptable long-term outcomes.

While the recommendation for surgical repair in AADA patients, even those who are intubated, aligns with current standard therapeutical strategies, our study offers crucial empirical support. It provides long-term outcome data for a highly fragile cohort of prehospital intubated AADA patients, demonstrating that despite high early mortality, an acceptable long-term outcome can be achieved. This evidence is vital for reinforcing the surgical approach in these critical ill patients, potentially alleviating hesitation among surgical teams and strengthening the rationale for current guidelines. Further multicenter studies are required to distinguish and evaluate the exact reasons for prehospital intubation in order to provide further information about the significantly increased mortality of this vulnerable cohort of prehospital intubated patients with AADA.

### Limitations

Due to the fact that this is a retrospective single-center study, it carries all the potential risks and biases associated with studies of this nature. A primary limitation is the inherent inconsistency of external prehospital records over the 18-year study period; while procedures followed DGAI guidelines, secondary numerical values to substantiate documented ‘hemodynamic instability’ or specific GCS scores were not consistently available for all patients. This variability limits the consistency of detailed intubation indications. Furthermore, given the relatively small sample size of our intubated cohort (n = 55), we deliberately avoided performing multivariable adjustment to prevent overfitting, which is a recognized limitation. The long study period (2000–2018) also means that surgical practices and perioperative management strategies, such as the adoption of the ‘beating heart’ technique or specific frozen elephant trunk (FET) approaches, evolved over time. While a temporal subgroup analysis would be ideal to account for this, the limited number of patients in the intubated cohort (*n* = 55) precludes further statistical comparisons across different eras, representing another inherent limitation of this retrospective study.

Finally, standardized long-term functional neurological scores (e.g., modified Rankin Scale) were not consistently available for the entire study period, limiting our ability to report on these outcomes comprehensively.

A patient’s outcome is undeniably associated with the factor of time. A large number of patients dying prior to admission can be expected.

## Conclusion

Prehospital intubated AADA patients represent a highly compromised and fragile subgroup within the broader AADA cohort. This group presents with a significantly higher burden of severe risk factors for early mortality, including pericardial tamponade, severe malperfusion, and preoperative resuscitation. We acknowledge that the decision for intubation is complex and highly individualized, reflecting the specific pathophysiological condition of each patient rather than a uniform indication. Our findings underscore that intubation in this context is a marker of critical illness. Despite the particularly high early mortality rate of approximately 40% in this severely ill subgroup, the majority of these patients still benefit from prompt surgical treatment. Furthermore, our study demonstrates an acceptable long-term outcome for patients who survive the initial year after treatment. Therefore, our data support the recommendation that emergent aortic repair should be actively considered and offered for intubated patients with AADA, recognizing the necessity for individualized management and the potential for favorable long-term survival in those who overcome the initial critical phase.

## Data Availability

No datasets were generated or analysed during the current study.
